# Supplementary material for: Fused Filament Fabrication of Slow-Crystallizing Polyaryletherketones: Crystallinity and Mechanical Properties Linked to Processing and Post-Treatment Parameters
Source: Polymers (Basel). 2024 Nov 29;16(23):3354. doi: 10.3390/polym16233354 (PMC11644239; doi:10.3390/polym16233354)
Supplement: Supplementary file 1 [file polymers-16-03354-s001.zip › polymers-3327085-supplementary.pdf]

Supporting Information

Fused Filament Fabrication of Slow-Crystallizing Polyaryletherketones: Crystallinity and Mechanical Properties Linked to Processing and Post-Treatment Parameters

Lucía Doyle [1], Xabier Pérez-Ferrero [2], Javier García-Molleja [1], Ricardo Losada [2], Pablo Romero-Rodríguez [2] and Juan P. Fernández-Blázquez [1,*]

[1] IMDEA Materials Institute, C/Eric Kandel, 2, Getafe, 28906 Madrid, Spain
[2] AIMEN Technology centre, O Porriño, 36418 Pontevedra, Spain

Table S1. Printer settings used in this work.

**Printing parameter**	**PEKK-A (X)**	**PEKK-A (Z)**	**AM200 (X)**	**AM200 (Z)**
**Slicer**	**KISSLICER**			
Nozzle temperature (°C)	370		385	
Build chamber temperature (°C)	90	80	75	75
Bed temperature (°C)	140		135	
Nozzle diameter (mm)	0.5			
Layer height (mm)	0.25			
Perimeter width (mm)	0.6			
Infill width (mm)	0.6	0.5	0.5	0.5
Flow	1	0.97	0.96	0.95
Perimeter infill overlap	1	0.75	1	0.75
Cooling fan (%)	0	100	50	100
Retraction	0			
Perimeter printing speed (mm s^{-1})	25			
Infill printing speed (mm s^{-1})	30			
Filament diameter (mm)	1.75			
Build plate material	Glass			
Adhesive	Nano polymer			

Figure S1. Crystallinity calculation for annealed samples using the 1D diffractograms.

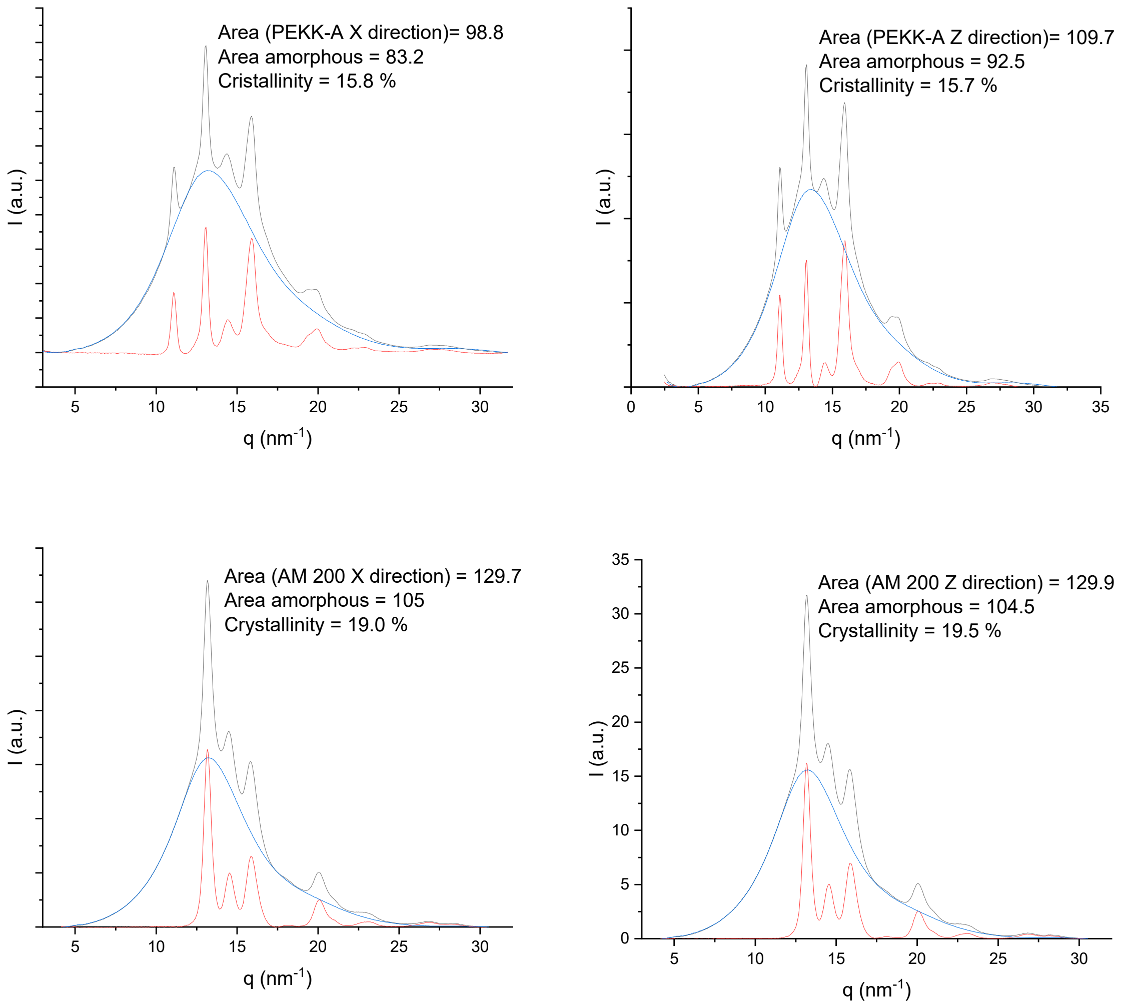

The calculation of crystallinity in semicrystalline samples (in our case, annealed ones) relates the areas of the narrow peaks (crystalline, red color) with the area of the pristine diffractogram (black line). In our case, the amorphous part (blue line) is obtained by normalizing amorphous samples from printed samples.
